# Effects of sgRNAs, Promoters, and Explants on the Gene Editing Efficiency of the CRISPR/Cas9 System in Chinese Kale

**DOI:** 10.3390/ijms241713241

**Published:** 2023-08-26

**Authors:** Wenli Huang, Aihong Zheng, Huanhuan Huang, Zhifeng Chen, Jie Ma, Xiangxiang Li, Qiannan Liang, Ling Li, Ruobin Liu, Zhi Huang, Yaoguo Qin, Yi Tang, Huanxiu Li, Fen Zhang, Qiaomei Wang, Bo Sun

**Affiliations:** 1 College of Horticulture, Sichuan Agricultural University, Chengdu 611130, China; 2021305054@stu.sicau.edu.cn (W.H.); zhengaihong@stu.sicau.edu.cn (A.Z.); hh820423@163.com (H.H.); 2021205028@stu.sicau.edu.cn (X.L.); 2022205029@stu.sicau.edu.cn (Q.L.); 201906195@stu.sicau.edu.cn (L.L.); 2022205026@stu.sicau.edu.cn (R.L.); huangzhi@sicau.edu.cn (Z.H.); 13185@sicau.edu.cn (Y.Q.); 13920@sicau.edu.cn (Y.T.); 10650@sicau.edu.cn (H.L.); zhangf@sicau.edu.cn (F.Z.); 2 College of Biology and Agricultural Technology, Zunyi Normal University, Zunyi 563006, China; czf810@163.com; 3 Bijie lnstitute of Agricultural Science, Bijie 551700, China; majie_011@126.com; 4 Department of Horticulture, Zhejiang University, Hangzhou 310058, China

**Keywords:** Chinese kale, CRISPR/Cas9, gene editing, sgRNAs, vectors, explants

## Abstract

The CRISPR/Cas9 system is extensively used for plant gene editing. This study developed an efficient CRISPR/Cas9 system for Chinese kale using multiple sgRNAs and two promoters to create various CRISPR/Cas9 vectors. These vectors targeted *BoaZDS* and *BoaCRTISO* in Chinese kale protoplasts and cotyledons. Transient transformation of Chinese kale protoplasts was assessed for editing efficiency at three *BoaZDS* sites. Notably, sgRNA: Z2 achieved the highest efficiency (90%). Efficiency reached 100% when two sgRNAs targeted *BoaZDS* with a deletion of a large fragment (576 bp) between them. However, simultaneous targeting of *BoaZDS* and *BoaCRTISO* yielded lower efficiency. Transformation of cotyledons led to Chinese kale mutants with albino phenotypes for *boazds* mutants and orange-mottled phenotypes for *boacrtiso* mutants. The mutation efficiency of 35S-CRISPR/Cas9 (92.59%) exceeded YAO-CRISPR/Cas9 (70.97%) in protoplasts, and YAO-CRISPR/Cas9 (96.49%) surpassed 35S-CRISPR/Cas9 (58%) in cotyledons. These findings introduce a strategy for enhancing CRISPR/Cas9 editing efficiency in Chinese kale.

## 1. Introduction

Chinese kale (*Brassica oleracea* var. *alboglabra*), a member of the Brassicaceae family, is an important vegetable in China. The main edible parts of Chinese kale are the flower heads and tender leaves, which are crisp and tender in texture, delicious in taste, and unique in flavor. Chinese kale is rich in carotenoids, vitamin C, and glucosinolates, making it highly nutritious and providing several health benefits [1]. Carotenoids are natural tetraterpene pigments that are widely distributed in plants, algae, fungi, and bacteria. These pigments bestow vibrant orange, yellow, and red hues on numerous flowers, fruits, and roots. After a series of desaturation and isomerization reactions catalyzed by phytoene desaturase (PDS), ζ-carotene desaturase (ZDS), ζ-carotene isomerase (Z-ISO), and carotenoid isomerase (CRTISO), the red carotenoid lycopene is formed from colorless phytoene. ZDS and CRTISO emerge as pivotal enzymes governing carotenoid accumulation in plants [2,3].

ZDS, as a rate-limiting enzyme involved in carotenoid synthesis, catalyzes the conversion of ζ-carotene to lycopene, making it a focal point of gene editing endeavors [4]. When the expression of the *ZDS* is suppressed, the chloroplasts of rice are deficient and whitened, resulting in seedling death [5], and maize seedlings exhibit an albino phenotype [6]. *Arabidopsis* deletion mutants show developmental retardation and an albino seedling phenotype due to severe carotenoids and chlorophyll deficiencies [7]; tomato leaves and fruits show photobleaching [8]. CRTISO is a key isomerase in the carotenoid biosynthetic pathway that converts yellow lycopene to red all-*trans* lycopene [9]. Loss of *CRTISO* function leads to the yellow color of several plant species. When *CRTISO* is down-regulated or silenced, melon seedlings turn yellow-green [10]; tomato fruits turn yellow [11]; rape petals turn yellow [12]; and the *CRTISO* mutant of Chinese kale changes color from green to yellow [13].

The CRISPR/Cas9 system is an RNA-guided endonuclease system consisting of Cas9 nuclease and customizable single-directed RNAs (sgRNAs), which guide the Cas9 endonuclease to complementary target DNA [14,15]. Cas9 nuclease cleaves double-stranded DNA at fixed sites to form double-stranded breaks (DSBs), which are repaired by homologous directional repair (HDR) and non-homologous terminal ligation (NHEJ). The HDR mechanism provides template DNA to correct mutations at DNA break sites, and the NHEJ mechanism randomly inserts or deletes nucleotides at the CRISPR-mediated double-stranded DNA break sites, which results in the destruction of gene-coding sequences [16]. The CRISPR/Cas9 system is a useful tool for editing target genes that can be used to rapidly improve plant traits; it allows one or more sequences to be targeted, thus greatly simplifying the gene editing process and broadening the selection range of the target site. CRISPR/Cas9 gene editing technology has been successfully applied to rice [17], wheat [18], maize [19], pear [20], strawberry [21], citrus [22], cucumber [23], rapeseed [24], etc.

The CRISPR/Cas9 system has been shown to be useful in various fields; however, its editing efficiency can be affected by several factors, including sgRNAs [25,26], the promoters of the editing vector [27,28], explants [29,30], etc. Further optimization through research and development is imperative. This study employed the CRISPR/Cas9 system to edit *BoaZDS* and *BoaCRTISO* in Chinese kale, employing various sgRNAs (single sgRNAs and double sgRNAs) and two promoters (*CaMV 35S* and *YAO*) to create diverse gene editing vectors. These vectors were introduced into Chinese kale protoplasts and cotyledons with petioles. The assessment of transgene-positive resistance rates and mutation rates underpins the exploration of factors influencing the editing system. This analysis aids in refining Chinese kale genome editing tools and contributes to characterizing gene functions and improving Chinese kale varieties in the future.

## 2. Results

### 2.1. Selection of the Target Site and Construction of the Vector

**Figure 1:**
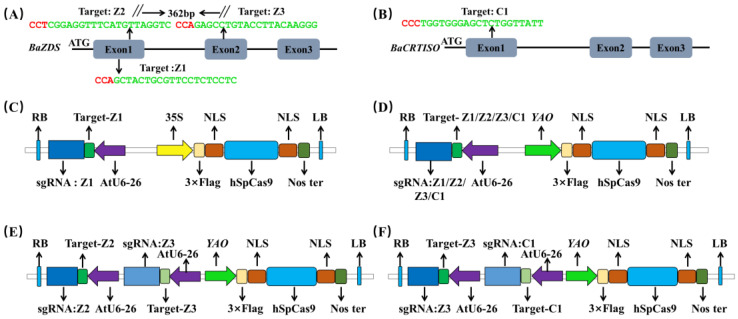
Selection of the target site and construction of the vector. (**A**) Schematic diagram of three target sites on *BoaZDS*. (**B**) Target site of *BoaCRTISO*. (**C**) sgRNA: Z1 was connected to the 35S-CRISPR/Cas9 vector. (**D**) sgRNA: Z1, sgRNA: Z2, sgRNA: Z3, or sgRNA: C1 was connected to the *YAO*-CRISPR/Cas9 vector. (**E**) sgRNA: Z2 and sgRNA: Z3, as well as (**F**) sgRNA: Z3 and sgRNA: C1, were connected in series and connected to the *YAO*-CRISPR/Cas9 vector, respectively.

To compare the editing efficiency of different vectors and target sites, we employed the 35S-CRISPR/Cas9 (CaMV 35S promoter-driven) and *YAO*-CRISPR/Cas9 (*YAO* promoter-driven) vectors in this experiment. Utilizing the online analysis tool (Supplementary Table S1), we selected three target sites (sgRNA: Z1, sgRNA: Z2, and sgRNA: Z3) from the first and second exons of *BoaZDS* (Figure 1A) and one target site, sgRNA: C1, from the first exon of *BoaCRTISO* (Figure 1B). sgRNA: Z1 was integrated into the 35S-CRISPR/Cas9 vector, while four target sites were integrated into the *YAO*-CRISPR/Cas9 vector (Figure 1C,D). By connecting sgRNA: Z2 and sgRNA: Z3, as well as sgRNA: Z3 and sgRNA: C1 in series, we attached the double-target sites to the *YAO*-CRISPR/Cas9 vector, respectively (Figure 1E,F). The constructed vectors were used for subsequent transformations.

### 2.2. Single sgRNA Targeting in the Transient Transformation of Protoplast

Four sgRNAs were selected to explore the effect of single sgRNAs on editing efficiency. 35S-CRISPR/Cas9 harboring sgRNA: Z1 was used to determine the optimal protoplast culture time. The highest mutation rate (92.59%) was achieved after 48 h of culture, surpassing rates after 24 h or 36 h. Consequently, 48 h of culture was adopted for subsequent protoplast culture (Table 1). When the sgRNA: Z1 was utilized with the *YAO*-CRISPR/Cas9 vector, the mutation rate after 48 h of culture was 70.97%, which is lower than 92.59%. This indicates that 35S-CRISPR/Cas9 exhibited superior editing efficiency compared to *YAO*-CRISPR/Cas9 during protoplast transient transformation (Table 1).

**Figure 2:**
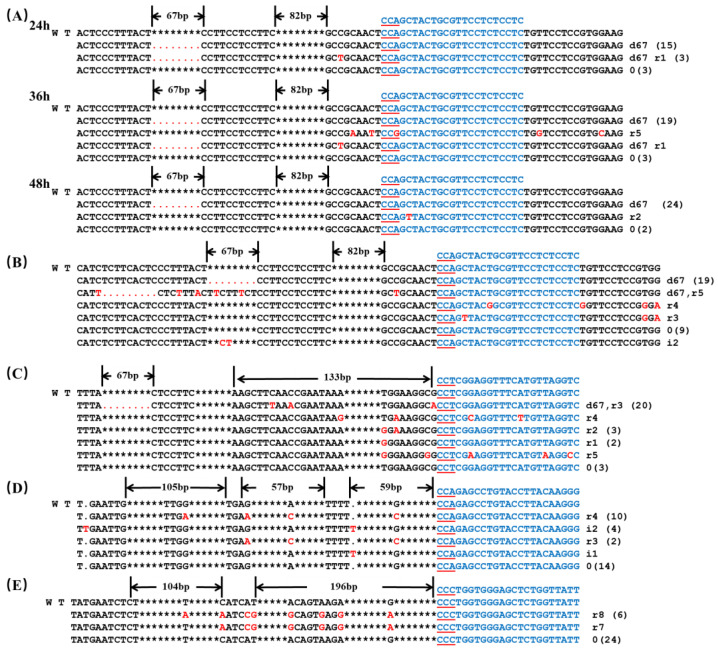
Mutation of protoplasts in Chinese kale. (**A**) Mutation of the 35S-CRISPR/Cas9 vector after protoplast culture at the sgRNA: Z1 target site for 24 h, 36 h, and 48 h. The mutation of the YAO- CRISPR/Cas9 vector at the target sites of (**B**) sgRNA: Z1, (**C**) sgRNA: Z2, (**D**) sgRNA: Z3, and (**E**) sgRNA: C1. WT: wild-type plant; the blue letters indicate the target sequence; the red underline indicates the PAM sequence; the red letters indicate the mutant base; the red dot indicates the missing base; * indicates the space between omitted bases; d indicates a base deletion; i indicates a base insertion; and r indicates a base substitution. The number after the sequence indicates the number of bases deleted, inserted, or replaced, and the number in parentheses indicates the number of clones.

Among the three different *BoaZDS* sgRNAs, the editing efficiency of sgRNA: Z2 was the highest (90%). In *BoaCRTISO*, the mutation rate of sgRNA: C1 was 22.58% (Table 1), which was much lower than the three *BoaZDS* sgRNA. After 48 h of culture, 25 clones were mutated, with 24 displaying a 67-bp fragment deletion upstream of sgRNA: Z1, and there was a base replacement at the target site, which was not observed in the other two groups (Figure 2A). The substantial 67-bp fragment deletion was observed in both target sites of sgRNA: Z1 and sgRNA: Z2. At sgRNA: Z1 and sgRNA: Z2, the target sequences of two clones were mutated, with one base replaced at sgRNA: Z1 and 2–3 bases replaced at sgRNA: Z2 (Figure 2B,C). For the target site sgRNA: Z3, only 2–4-bp single-base replacements were observed upstream of the target site without large fragment deletions (Figure 2D). Consequently, considering the mutation rate and target sequence mutation, sgRNA: Z2 had the highest editing efficiency among the three sgRNAs. At the target site sgRNA: C1, only 7–8-bp replacements occurred upstream of the target site, devoid of base deletion or insertion (Figure 2E); this exhibited low editing efficiency.

### 2.3. Double sgRNA Targeting in the Transient Transformation of the Protoplast

**Figure 3:**
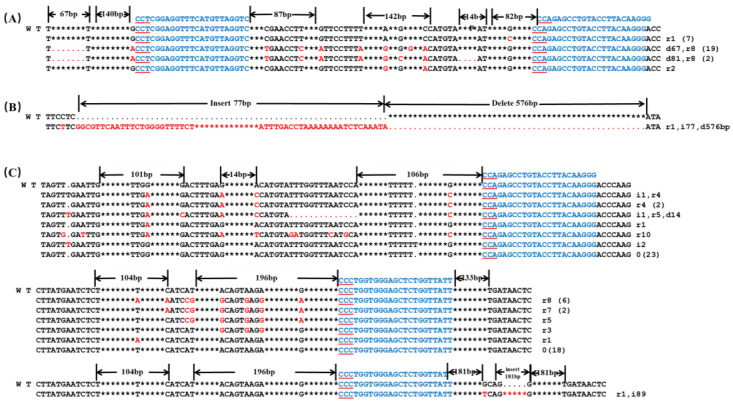
Mutation of the double-target site in Chinese kale. (**A**,**B**) Mutations of the double-target site sgRNA: Z2-Z3 in the *BoaZDS* gene. (**C**) Mutations at the double-target site of sgRNA: Z3-C1 in the *BoaZDS* and *BoaCRTISO* genes. WT: wild-type plant; the blue letters indicate the target sequence; the red underline indicates the PAM sequence; the red letters indicate the mutant base; the red dot indicates the missing base; * indicates the space between omitted bases; d indicates a base deletion; i indicates a base insertion; and r indicates a base substitution. The number after the sequence indicates the number of bases deleted, inserted, or replaced, and the number in parentheses indicates the number of clones.

Double target sites (sgRNA: Z2-Z3) were selected for editing *BoaZDS*, and they were induced using the *YAO*-CRISPR/Cas9 vector. All 30 clones exhibited mutations in the protoplast transient transformation (Table 2), indicating that the double target’s editing efficiency (100%) surpassed that of individual single targets within *BoaZDS*. Mutations with 67-bp deletions and single-base replacements preceding the target site, along with the deletion of a large fragment (576 bp), were also detected (Figure 3A,B).

Two sgRNAs (sgRNA: Z3-C1) from *BoaZDS* and *BoaCRTISO* were chosen to edit double-target genes, with a mutation rate of 23.33% for *BoaZDS* and 40% for *BoaCRTISO* (Table 2). A low mutation efficiency was observed when two distinct genes were concurrently edited by double sgRNAs. In *BoaZDS*, only one clone had a 14-bp deletion upstream of sgRNA: Z1, while *BoaCRTISO* exhibited an 89-bp insertion. The number of remaining mutated bases was 1–10 bp, all of which were single-base substitutions or deletions (Figure 3C). These results suggest that enhancing editing efficiency might occur when a single gene is edited by two sgRNAs, possibly resulting in large deletions. However, when two sgRNAs simultaneously edit two genes, the editing efficiency decreases.

### 2.4. Stable Genetic Transformation of BoaZDS in Chinese Kale Cotyledons with Petioles

The positive transformation rate of 35S-CRISPR/Cas9 at the sgRNA: Z1 target site was 96.15% (Supplementary Figure S1), while for YAO-CRISPR/Cas9, it was 98.21% at sgRNA: Z1 and 100% at sgRNA: Z2 (Supplementary Figures S2 and S3). A total of 50 transgenic positive plants were obtained by 35S-CRISPR/Cas9 editing with sgRNA: Z1, with a mutation rate of 58% (Supplementary Table S2). In contrast, *YAO*-CRISPR/Cas9 achieved a mutation efficiency of 96.49% in site-directed editing using sgRNA: Z1, which was approximately 1.7 times higher than 35S-CRISPR/Cas9. The mutation rate of sgRNA: Z2 was 80%, slightly lower than that of sgRNA: Z1 (Supplementary Table S2).

**Figure 4:**
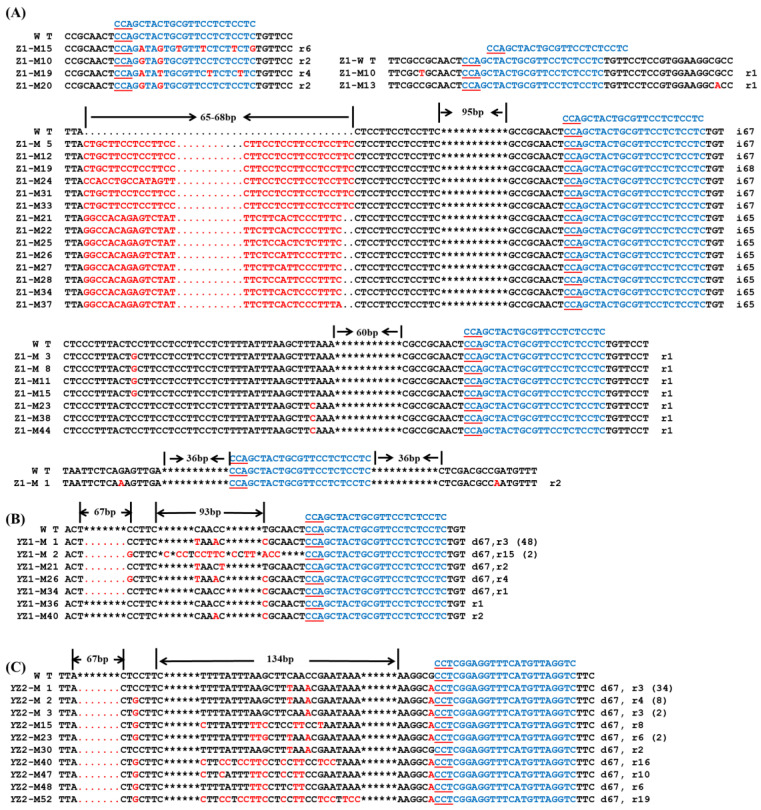
Mutation of the *BoaZDS* gene in Chinese Kale. (**A**) Mutation of the 35S-CRISPR/Cas9 vector at the sgRNA: Z1 target site. The mutation of the *YAO*- CRISPR/Cas9 vector at the target sites of (**B**) sgRNA: Z1 and (**C**) sgRNA: Z2. Z1-M1 indicates a mutant of 35S-CRISPR/Cas9 at sgRNA: Z1, and YZ1-M1 indicates a mutant of *YAO*-CRISPR/Cas9 at sgRNA: Z1. The blue letters indicate the target sequence; the red underline indicates the PAM sequence; the red letters indicate the mutant base; the red dot indicates the missing base; * indicates the space between omitted bases; d indicates a base deletion; i indicates a base insertion; r indicates a base substitution. The number after the sequence indicates the number of bases deleted, inserted, or replaced, and the number in parentheses indicates the number of mutants.

**Figure 5:**
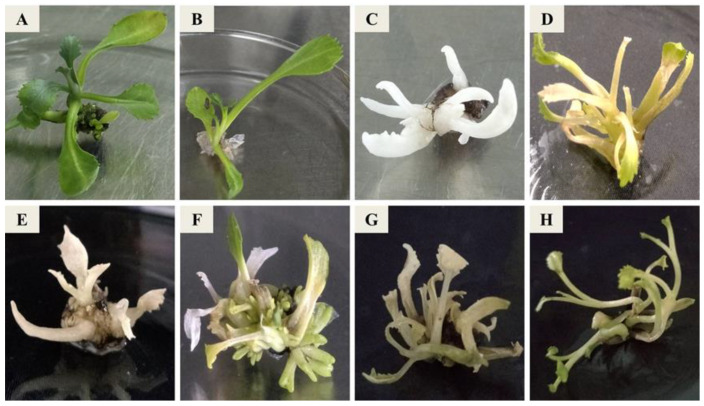
Phenotype of *zds* mutants in Chinese kale. The letters in the upper left corner of the picture indicate different mutants. (**A**): wild type; (**B**): transgenic plant with empty vector; (**C**): Z1-M15; (**D**): Z1-M5; (**E**): Z1-M3; (**F**): Z1-M1; (**G**): YZ1-M34; (**H**): YZ2-M40.

A total of 29 mutants were obtained via 35S-CRISPR/Cas9 editing of sgRNA: Z1. Among them, 15 displayed a large fragment deletion of 65–68 bp, while the remaining 14 plants exhibited single-base substitutions of 1–6 bp (Figure 4A). When *YAO*-CRISPR/Cas9 was used to perform gene editing, 55 mutants were obtained at sgRNA: Z1. Within this group, 53 mutants exhibited a 67-bp fragment deletion and a 1–15-bp single-base substitution. Additionally, two mutants show a 1–2-bp single-base substitution (Figure 4B). A total of 52 mutants were obtained via editing of sgRNA: Z2, including 67-bp fragment deletions and single-base substitutions, with up to 19 base substitutions observed (Figure 4C). These mutants presented either a pure albino phenotype or a mosaic albino phenotype (Figure 5).

### 2.5. Stable Genetic Transformation of BoaCRTISO in Chinese Kale Cotyledons with Petioles

A total of 17 resistant plants were detected after stable genetic transformation, and the positive resistance rate was 100% (Supplementary Figure S4). Following sequence analysis of transgenic positive strains, 15 mutants were obtained, reflecting a mutation rate of 88.24% (Supplementary Table S2). Within these mutants, 47.06% were heterozygous mutations, while 41.18% were chimeric mutations (Supplementary Table S3).

**Figure 6:**
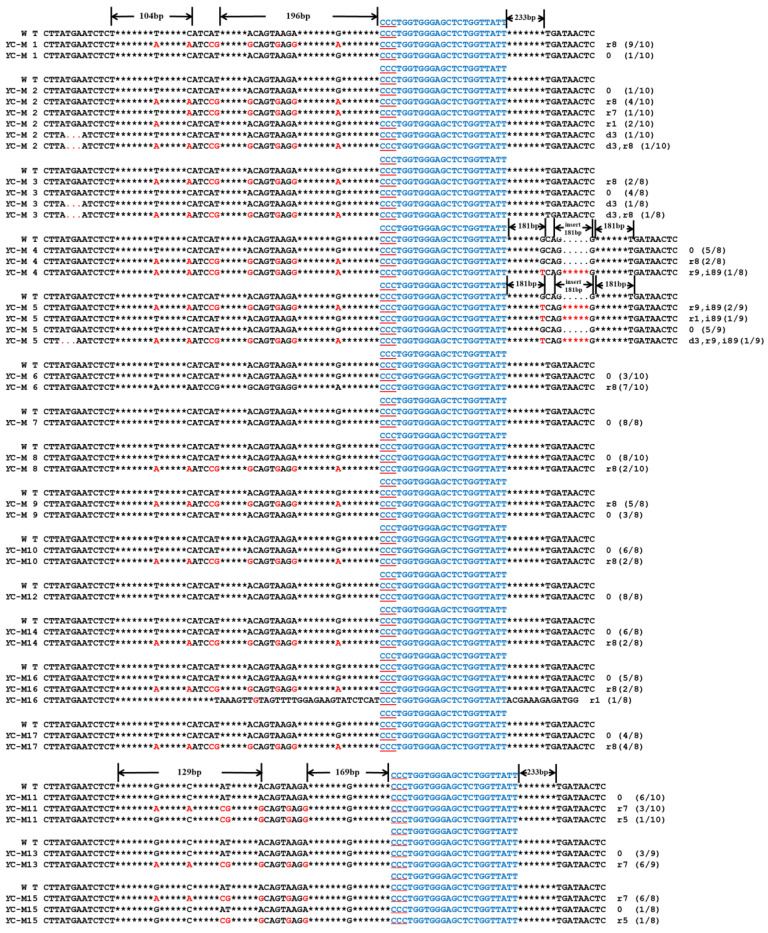
Mutation of the *BoaCRTISO* gene in Chinese kale. YC1-M1 indicates a mutant of the *YAO*-CRISPR/Cas9 vector at sgRNA: C1; the blue letters indicate the target sequence; the red underline indicates the PAM sequence; the red letters indicate the mutant base; the red dot indicates the missing base; * indicates the space between omitted bases; d indicates a base deletion; i indicates a base insertion; and r indicates the base substitution. The number after the sequence indicates the number of bases deleted, inserted, or replaced, and the number in parentheses indicates the number of mutants.

**Figure 7:**
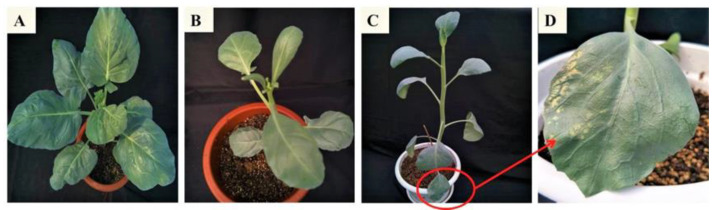
Phenotype of *crtiso* mutants in Chinese kale. (**A**): wild type; (**B**): transgenic plant with empty vector; (**C**,**D**): YC-M11.

Except for the substitution of 1–9 single bases, mutants YC-M4 and YC-M5 featured 89-bp fragments inserted after the target site, whereas YC-M2, YC-M3, and YC-M5 displayed three consecutive base deletions upstream of the target site (Figure 6). Notably, one mutant (YC-M11) exhibited an orange-mottled phenotype (Figure 7). There was no obvious difference in growth and development between mutants and wild-type plants.

## 3. Discussion

In this study, we employed a combination of multiple sgRNAs and two promoters to construct different gene editing vectors to edit *BoaZDS* and *BoaCRTISO*. Using the CRISPR/Cas9 system, we successfully transformed Chinese kale protoplasts and cotyledons with petioles. Sequencing analysis confirmed gene editing, and the corresponding mutants were generated.

### 3.1. Effect of sgRNA on the Editing Efficiency of the CRISPR/Cas9 System

Several studies have highlighted sgRNA as the most important factor affecting editing efficiency [31]. To edit *BoaZDS*, three sgRNAs were selected using an online analysis tool. The editing efficiency of sgRNA: Z2 was the highest (90%) in protoplasts; however, its efficiency in cotyledons with petioles was lower than sgRNA: Z1. Previous research indicates a connection between CRISPR/Cas9 sgRNA activity and GC content, as well as the protospacer adjacent motif (PAM) site [32]. Editing efficiency is higher when the GC content exceeds 50%, while lower GC content (40%) corresponds to reduced efficiency [33]. Modifying Cas9 has been explored to heighten PAM affinity and bolster genome editing efficacy [34].

### 3.2. Effect of Double-sgRNAs on the Editing Efficiency of the CRISPR/Cas9 System

In recent years, plant editing vectors have incorporated multiple sgRNAs targeting multiple genes. The CRISPR/Cas9 system has been designed to simultaneously edit multiple genes in plants, allowing systematic exploration of gene families and metabolic pathways [35,36]. In the protoplast transient transformation, the *YAO*-CRISPR/Cas9 vector enabled simultaneous editing of two *BoaZDS* target sites, achieving 100% editing efficiency. The deletion of a 576-bp fragment was observed, likely greatly affecting gene function and enhancing plant traits. The editing efficiency was improved when two sgRNAs were employed for *BoaZDS* editing. Meanwhile, when two sgRNAs were used to edit two genes, the mutation rate of *BoaCRTISO* was 40%, nearly doubling that of a single sgRNA, and an 89-bp fragment deletion was observed. However, in *BoaZDS*, the editing efficiency was approximately half that of the single sgRNA, with no large fragment deletions. This indicates that connecting two sgRNAs in the CRISPR/Cas9 system does not significantly enhance the efficiency of editing two genes, and similar results have been observed in recent studies [37].

### 3.3. Effect of Promoters on the Editing Efficiency of the CRISPR/Cas9 System

The promoter plays a pivotal role in driving Cas9 expression within the vector. The cauliflower mosaic virus 35S promoter (CaMV 35S) is widely used to induce gene expression in a variety of plant species [38]. In dicotyledonous plants, the mutation frequency ranges from 26% to 95% with the use of the CaMV 35S promoter, aligning with the mutation frequencies observed in our study [33]. Introducing a species-specific promoter for Cas9 expression can greatly increase the mutation frequency. For instance, when the *YAO* promoter was used to drive CRISPR/Cas9 expression, the Cas9 gene editing efficiency significantly increased [39]. In our study, the 35S promoter and the *YAO* promoter were used for 35S-CRISPR/Cas9 and *YAO*-CRISPR/Cas9, respectively. They were linked to sgRNA: Z1 to form two different binary vectors. The mutation efficiency of 35S-CRISPR/Cas9 and *YAO*-CRISPR/Cas9 was inconsistent, underscoring the impact of promoter diversity on Cas9 expression and, in turn, the gene editing efficiency. The 35S-CRISPR/Cas9 vector had a higher mutation efficiency in protoplasts, while the *YAO*-CRISPR/Cas9 vector had a greater mutation efficiency in cotyledons with petioles. This points to elevated *Cas9* expression driven by the *YAO* promoter in tissues with heightened cell division, but lower levels in protoplasts [40]. The results indicated that selecting an appropriate promoter can improve the editing efficiency of the CRISPR/Cas9 system in Chinese kale.

### 3.4. Effect of Explants on the Editing Efficiency of the CRISPR/Cas9 System

To explore the effect of different explants on editing efficiency, the protoplasts and cotyledons with petioles of Chinese kale were employed for editing. Plant protoplasts can offer a convenient means to evaluate and refine gene editing tools [37,41]. Before transforming plants, we first transformed the protoplasts of Chinese kale to verify the effectiveness of the selected gene editing targets. Mutants were obtained by transforming cotyledons with petioles. Interestingly, the gene editing efficiency exhibited variation between protoplasts and cotyledons with petioles using the same vector, suggesting that the explants can affect the editing efficiency. In the stable genetic transformation of cotyledons with petioles, no homozygous or biallelic mutations in *BoaCRTISO* were observed. It is worth noting that transformed explants often exhibit chimerism, particularly in dicotyledonous plants [42]. Yet, chimerism occurrence is lower during protoplast regeneration [41]. Consequently, the selection of explants for transformation is also an important factor affecting the efficiency of gene editing.

## 4. Materials and Methods

### 4.1. Plant Material and Cultivation Conditions

Aseptic seedlings of the ‘Sijicutiao’ white Chinses kale cultivar were grown in medium containing Murashige and Skoog (1/2 MS) at 25 °C on a rack with a photoperiod of 16 h and a light intensity of 36 μmol m^−2^ s^−1^.

### 4.2. Selection of sgRNAs and Construction of the CRISPR/Cas9 Vector

The online analysis tool (http://crispr.hzau.edu.cn (accessed on 5 January 2022)) was used to analyze the gene sequences, and a total of four target sites of *BoaZDS* and *BoaCRTISO* were selected (Supplementary Table S1). The primers required for constructing the vector are shown in Supplementary Table S4. Vectors were constructed following the methods described in previous studies [39]. The *Arabidopsis* U6 promoter *AtU6-26* was used to drive the expression of sgRNAs, and the CaMV 35S promoter and *YAO* promoter were used to drive the expression of *Cas9* in the 35S-CRISPR/Cas9 and *YAO*-CRISPR/Cas9 vectors, respectively. The primers sgRNA: Z1-F/R, sgRNA: Z2-F/R, sgRNA: Z3-F/R, and sgRNA: C1-F/R were synthesized, and the annealed primers were mixed with *Bsa*I-digested AtU6-26-sgRNA-SK plasmids. In the construction of the double targets, one of the sgRNA cassette vectors was digested by *Spe*I, and then the other sgRNA cassette was digested by *Nhe*I and *Spe*I; the recovered fragments were connected to the sgRNA cassette vector digested by *Spe*I by T4 DNA ligase to form the double gRNA cassette vector. The *YAO*: Cas9 and 35S: Cas9 vectors were digested by *Spe*I and ligated to the resulting sgRNA cassette for transient and genetic transformation, respectively (Figure 1).

### 4.3. PEG-Mediated Transient Transformation of Chinese Kale Protoplasts

Chinese kale protoplasts were transformed following methods described in previous studies [43]. For the transient expression of protoplasts, the prepared protoplasts were placed on ice and left to settle for 30 min, and most of the supernatant was dried. MMG solution was added at a specific concentration (5–8 × 10^5^) cells/mL, and 100 μL of protoplasts were transferred to a 2 mL centrifuge tube. After 10 μg of the plasmid to be converted was added, it was mixed by flicking. Next, 100 μL of PEG transfection solution was added and mixed gently. The mixture was allowed to stand at room temperature for 15 min under dark conditions, and 550 μL of W5 solution was added to stop the reaction. The mixture was then mixed by turning the tube upside down. After centrifugation at 100× *g* for 2 min, the precipitate was suspended in 1 mL of WI solution. After the protoplasts were cultured at approximately 23 °C for 24 h, 36 h, and 48 h in the dark, the optimal transformation time was determined to be 48 h. Protoplast DNA was extracted using a DNA kit.

### 4.4. Agrobacterium-Mediated Stable Transformation of Chinese Kale Cotyledons with Petioles

The transformation of Chinese kale was performed as described in our previous study [44]. The sterilized seeds were sown on a 1/2 MS culture medium, cultured at 25 °C for 3 days under dark conditions, and then grown on the medium for 3 to 4 days under 16 h light/8 h dark conditions. The sessile cotyledons with 1–2 mm long petioles were cut from the aseptic kale seedling. The cotyledons with petioles were incubated in pre-medium (MS + 0.5 mg·L^−1^ 2,4-Dichlorophenoxyacetic acid (2,4-D) + 2% sucrose + 0.8% agar) for 2–3 days. Then, they were disseminated in Agrobacterium (GV3101) bacterial solution for 1–2 min and transferred to co-culture medium (MS + 0.03 mg·L^−1^ naphthaleneacetic acid (NAA) + 0.75 mg·L^−1^ 6-Benzylaminopurine (6-BA) + 2% sucrose + 0.8% agar) for 2–3 days, and then transferred into a delayed screen medium (MS + 0.03 mg·L^−1^ NAA + 0.75 mg·L^−1^ 6-BA + 320 mg·L^−1^ Carbenicillin + 320 mg·L^−1^ Timentin + 2% sucrose + 0.8% agar for 7 days, and then transferred into a resistance screening medium (MS + 0.03 mg·L^−1^ NAA + 0.75 mg·L^−1^ 6-BA + 320 mg·L^−1^ Carbenicillin + 320 mg·L^−1^ Timentin + 12 mg·L^−1^ hygromycin B (Hyg) + 2% sucrose + 0.8% agar) to delay screening.

### 4.5. Detection of Mutations

Genomic DNA from protoplasts, hygromycin-resistant plants, and wild-type plants was extracted. To evaluate the transformation efficiency, specific primers, Hyg-F/R, were used to amplify the genomic DNA. To evaluate the mutation efficiency, the target sites of the protoplast and positive bud were amplified separately using the primers sgRNA: Z1-CRISPR test-F/R, sgRNA: Z2-CRISPR test-F/R, sgRNA: Z3-CRISPR test-F/R, and sgRNA: C1- CRISPR test-F/R, and sequenced to determine the presence of mutations (Supplemental Table S4). Approximately 20–30 clones were picked from each target site of the protoplasts, and 8–10 clones were picked from each transgenic positive bud for sequencing to calculate the mutation rate, collect all sequencing data, analyze the mutation type of the mutants, and take photos.

## 5. Conclusions

The results of our study indicate that sgRNAs affected editing efficiency, and the factors affecting the efficiency of sgRNAs were mainly related to the GC content and PAM sites. Different promoters affect the activity of Cas9; the *35S* promoter had higher activity in Chinese kale protoplasts, and the *YAO* promoter had higher activity in cotyledons with petioles; thus, the selection of appropriate promoters can help improve the efficiency of the vector. The efficiency of the same vector in different explants also varied; thus, the selection of the editing explants is critically important. In this study, we characterized the effects of different factors on the efficiency of the CRISPR/Cas9 system, which was helpful to improve the gene editing efficiency of Chinese kale and to carry out targeted genetic improvement on Brassica vegetables, thus speeding up the breeding process of Brassica vegetables and cultivating more new varieties of Brassica vegetables with dominant characters.

## Figures and Tables

**Table 1:** Editing efficiency of single sgRNAs in Chinese kale protoplasts.

CRISPR/Cas9 Vector	Target Gene	Target Site	Cultivate Time (h)	Number of Clones Detected	Number of Mutant Clones	Mutation Rate (%)
35S-CRISPR/Cas9	*BoaZDS*	sgRNA: Z1	24	21	18	85.71
			36	24	21	87.5
			48	27	25	92.59
YAO-CRISPR/Cas9	*BoaZDS*	sgRNA: Z1	48	31	22	70.97
		sgRNA: Z2	48	30	27	90
		sgRNA: Z3	48	31	17	54.84
	*BoaCRTISO*	sgRNA:C1	48	31	7	22.58

**Table 2:** Editing efficiency of double sgRNAs in protoplasts.

Target Site	Target Gene	Number of Clones Detected	Number of Mutant Clones	Mutation Rate (%)
sgRNA: Z2-Z3	*BoaZDS*	30	30	100
sgRNA: Z3-C1	*BoaZDS*	30	7	23.33
	*BoaCRTISO*	30	12	40

## Data Availability

All data supporting the findings of this study are available within the paper and within its Supplementary Materials published online.

## Supplementary Materials

The following supporting information can be downloaded at: https://www.mdpi.com/article/10.3390/ijms241713241/s1.
