# Supplementary material for: Effects of sgRNAs, Promoters, and Explants on the Gene Editing Efficiency of the CRISPR/Cas9 System in Chinese Kale
Source: Int J Mol Sci. 2023 Aug 26;24(17):13241. doi: 10.3390/ijms241713241 (PMC10487834; doi:10.3390/ijms241713241)
Supplement: Supplementary file 1 [file ijms-24-13241-s001.zip › Figure S2. Hyg resistance test of the YAO- CRISPR-Cas9 vector at the sgRNA Z1 target site.pdf]

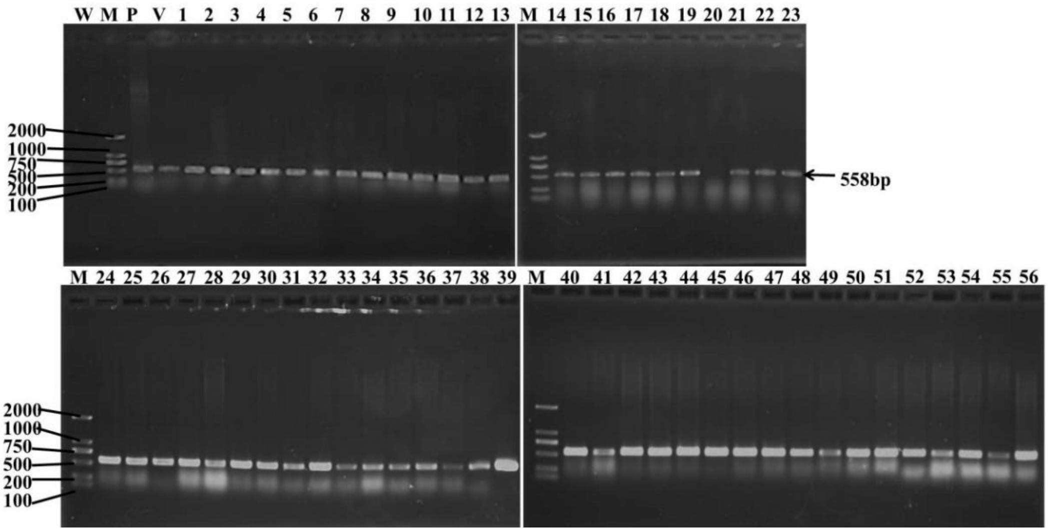

Figure S2. Hyg resistance test of the YAO- CRISPR/Cas9 vector at the sgRNA: Z1 target site. W: water as negative control, M: DL2000 marker, P: positive plasmid, V: empty vector, 1-56: the number resistant plants.
