# Supplementary material for: Effects of sgRNAs, Promoters, and Explants on the Gene Editing Efficiency of the CRISPR/Cas9 System in Chinese Kale
Source: Int J Mol Sci. 2023 Aug 26;24(17):13241. doi: 10.3390/ijms241713241 (PMC10487834; doi:10.3390/ijms241713241)
Supplement: Supplementary file 1 [file ijms-24-13241-s001.zip › Table S1. The GC content of target sites.pdf]

Table S1. The GC content of target sites.

Name of Target site	Target site sequence	sgRNA GC content
sgRNA: Z1	CCAGCTACTGCGTTCCTCTCCTC	60%
sgRNA: Z2	CCTCGGAGGTTTCATGTTAGGTC	50%
sgRNA: Z3	CCAGAGCCTGTACCTTACAAGGG	55%
sgRNA: C1	CCCTGGTGGGAGCTCTGGTTATT	50%
