# Supplementary material for: Effects of sgRNAs, Promoters, and Explants on the Gene Editing Efficiency of the CRISPR/Cas9 System in Chinese Kale
Source: Int J Mol Sci. 2023 Aug 26;24(17):13241. doi: 10.3390/ijms241713241 (PMC10487834; doi:10.3390/ijms241713241)
Supplement: Supplementary file 1 [file ijms-24-13241-s001.zip › Table S2. Mutation rate of BoaZDS and BoaCRTISO in Chinese kale.pdf]

Table S2. Mutation rate of *BoaZDS* and *BoaCRTISO* in Chinese kale.

CRISPR/Cas9 Vector	Target gene	Target site	Number of plants tested	Number of mutants	Mutation rate (%)
35S-CRISPR/Cas9	*BoaZDS*	sgRNA: Z1	50	29	58
YAO-CRISPR/cas9	*BoaZDS*	sgRNA: Z1	57	55	96.49
	BoaZDS	sgRNA: Z2	65	52	80
	BoaCRTISO	sgRNA: C1	17	15	88.24
