# Supplementary material for: Effects of sgRNAs, Promoters, and Explants on the Gene Editing Efficiency of the CRISPR/Cas9 System in Chinese Kale
Source: Int J Mol Sci. 2023 Aug 26;24(17):13241. doi: 10.3390/ijms241713241 (PMC10487834; doi:10.3390/ijms241713241)
Supplement: Supplementary file 1 [file ijms-24-13241-s001.zip › Table S3. Mutation types of BoaCRTISO in Chinese kale.pdf]

Table S3. Mutation types of *BoaCRTISO* in Chinese kale.

Mutation type	Bi-allelic	Homozygous	Heterozygous	Chimeric	WT
No. of plants with mutations	0	0	8	7	2
Mutation rate (%)	0	0	47.06	41.18	11.76
