# Supplementary material for: Effects of sgRNAs, Promoters, and Explants on the Gene Editing Efficiency of the CRISPR/Cas9 System in Chinese Kale
Source: Int J Mol Sci. 2023 Aug 26;24(17):13241. doi: 10.3390/ijms241713241 (PMC10487834; doi:10.3390/ijms241713241)
Supplement: Supplementary file 1 [file ijms-24-13241-s001.zip › Table S4. Primers were used in this study.pdf]

Table S4. Primers were used in this study.

Primer names	Sequence of primers (5'-3')	Aims
sgRNA: Z1-F	ATTGAGGAGAGGAACGCAGTAGC	The synthesis of target site
sgRNA: Z1-R	AAACGCTACTGCGTTCCTCTCCT	
sgRNA: Z2-F	ATTGACCTAACATGAAACCTCCG	
sgRNA: Z2-R	AAACCGGAGGTTTCATGTTAGGTC	
sgRNA: Z3-F	ATTGGAGCCTGTACCTTACAAGGG	
sgRNA: Z3-R	AAACCCTTGTAAGGTACAGGCTCT	
sgRNA: C1-F	ATTGAATAACCAGAGCTCCCACCA	
sgRNA: C1-R	AAACTGGTGGGAGCTCTGGTTATT	
sgRNA: Z1-CRISPR test-F	ATCCCACTGGCCATAGTTAGGC	Detection of the mutation in transgenic plants
sgRNA: Z1-CRISPR test-R	CAAGTCCAGCTCCAATGATAGCTAC	
sgRNA: Z2-CRISPR test-F	ATCCCACTGGCCATAGTTAGGC	
sgRNA: Z2-CRISPR test-R	CAAGTCCAGCTCCAATGATAGCTAC	
sgRNA: Z3-CRISPR test-F	CTCTCGACGCCGATGTTTCCG	
sgRNA: Z3-CRISPR test-R	CATCAAGCGGAAGAGGTTGTTGTAG	
sgRNA: C1- CRISPR test-F	ATGAATCTCTGTCTCCGCAACCC	
sgRNA: C1-CRISPR test-R	AACCTCCATCTCACGACCAACTG	
Hyg-F	CGATTGCGTCGCATCGACC	Detection of the hygromycin resistance gene
Hyg-R	TTCTACAACCGGTCGCGGAG	
